# Identification of three new *cis*-regulatory *IRF5 *polymorphisms: *in vitro *studies

**DOI:** 10.1186/ar4262

**Published:** 2013-08-13

**Authors:** Elisa Alonso-Perez, Romina Fernandez-Poceiro, Emilie Lalonde, Tony Kwan, Manuel Calaza, Juan J Gomez-Reino, Jacek Majewski, Antonio Gonzalez

**Affiliations:** 1 Laboratorio Investigacion 10 and Rheumatology Unit, Instituto de Investigacion Sanitaria, Hospital Clinico Universitario de Santiago, Travesia Choupana, s/n. Santiago de Compostela 15706, Spain; 2 Department of Human Genetics, McGill University, Stewart Biology Building, 1205 Dr Penfield Avenue, Montreal, QC, H3A 1B1, Canada; 3 Department of Medicine, University of Santiago de Compostela, Rúa de San Francisco, s/n. Santiago de Compostela 15782, Spain

## Abstract

**Background:**

Polymorphisms in the interferon regulatory factor 5 (*IRF5*) gene are associated with susceptibility to systemic lupus erythematosus, rheumatoid arthritis and other diseases through independent risk and protective haplotypes. Several functional polymorphisms are already known, but they do not account for the protective haplotypes that are tagged by the minor allele of rs729302.

**Methods:**

Polymorphisms in linkage disequilibrium (LD) with rs729302 or particularly associated with *IRF5 *expression were selected for functional screening, which involved electrophoretic mobility shift assays (EMSAs) and reporter gene assays.

**Results:**

A total of 54 single-nucleotide polymorphisms in the 5' region of *IRF5 *were genotyped. Twenty-four of them were selected for functional screening because of their high LD with rs729302 or protective haplotypes. In addition, two polymorphisms were selected for their prominent association with *IRF5 *expression. Seven of these twenty-six polymorphisms showed reproducible allele differences in EMSA. The seven were subsequently analyzed in gene reporter assays, and three of them showed significant differences between their two alleles: rs729302, rs13245639 and rs11269962. Haplotypes including the *cis*-regulatory polymorphisms correlated very well with *IRF5 *mRNA expression in an analysis based on previous data.

**Conclusion:**

We have found that three polymorphisms in LD with the protective haplotypes of *IRF5 *have differential allele effects in EMSA and in reporter gene assays. Identification of these *cis*-regulatory polymorphisms will allow more accurate analysis of transcriptional regulation of *IRF5 *expression, more powerful genetic association studies and deeper insight into the role of *IRF5 *in disease susceptibility.

## Introduction

The interferon regulatory factor 5 (*IRF5*) gene occupies a prominent place among the genetic factors involved in susceptibility to rheumatic and autoimmune diseases [1]. It is associated with a large series of diseases, either definitively, as with systemic lupus erythematosus (SLE) [2-7], Sjögren's syndrome, systemic sclerosis, primary biliary cirrhosis and rheumatoid arthritis; or more tentatively, as with granulomatosis with vasculitis, multiple sclerosis, inflammatory bowel disease and atherosclerosis [1]. Discovery of these associations has contributed to increased interest in the role of type I interferon in autoimmune diseases and to progress in understanding of disease pathogenesis, particularly regarding SLE [8,9]. However, advances could be made faster if we understood the molecular and cellular mechanisms involved. A variety of functional polymorphisms have already been identified in the *IRF5 *locus, but their relationship with disease susceptibility is still unclear.

Four polymorphisms with a putative functional role have been described [2-4,7,10,11]. The first identified is rs2004640, in which the T allele introduces a donor splice site for alternative first exons [3]. However, its *in vivo *relevance has been questioned [7]. Another, rs10954213, creates an early polyadenylation site that leads to shorter *IRF5 *mRNA isoforms with longer half-lives [4]. This single-nucleotide polymorphism (SNP) has the strongest evidence for a role in *cis*-regulatory element of *IRF5 *[12,13]. The remaining two functional polymorphisms are of the insertion-deletion (indel) type. One of them, with 3x or 4x copies of CGGGG in the *IRF5 *promoter, affects transcription levels by differential binding of the specificity protein 1 (Sp1) transcription factor [10,11]. The other changes 10 amino acids encoded in exon 6, but experimental evidence of any effect of this change in the IRF5 protein is still lacking [7].

Various models have been proposed to account for the association with disease in this locus, including combinations of the functional polymorphisms [4-7,11], but none is completely satisfactory. In particular, haplotypes with two opposed effects have consistently been observed in the association of *IRF5 *with SLE [4-7] and with other diseases [14-17]. However, we still do not know the causes of the two effects. Most studies have focused on the risk haplotype (designated as haplotype 6 in [5,12,15] and described in Additional file 1: Note S1), which has a frequency of about 10% in controls and nearly 20% in SLE patients. This haplotype includes alleles that determine increased expression of the gene (the 4x allele of the CGGGG indel in the promoter) and longer half-life of the mRNA (the A allele of rs10954213 in the 3' untranslated region). However, it is unclear whether increased expression is the only mechanism involved, because there are other haplotypes that include combinations of the same alleles but are not associated with increased SLE risk (for example, haplotypes 4 and 5, which include alleles 4x and A but are neutral). It has been proposed that the difference between the risk and the neutral haplotypes resides in interactions with other functional polymorphisms, such as the indel in exon 6, but this hypothesis lacks experimental support. Less effort has been put into investigating the protective effect of IRF5 variants. In multiple studies, disease protection is associated with the minor allele of rs729302 [4-6], which is a SNP of unapparent functionality located 5' to the gene, and with two haplotypes (designated as haplotypes 1 and 2 in our studies; see Additional file 1: Note S1). This protective effect does not correlate with the known functional polymorphisms. Therefore, we currently lack any explanation for disease protection to account for *cis *polymorphisms in the *IRF5 *locus.

**Figure 1:**
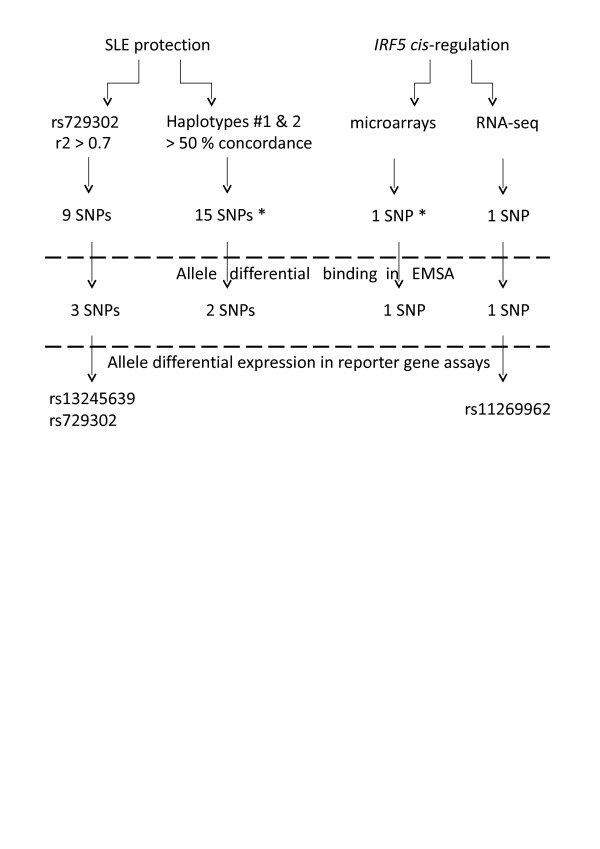
**Experimental design of the search for new functional polymorphisms in *IRF5***. Polymorphisms for functional screening were selected on the basis of correlation with systemic lupus erythematosus (SLE) protection or prominent role is *IRF5 cis*-regulation models. A first functional screen by electrophoretic mobility shift assay (EMSA) was applied to the 26 selected polymorphisms, and a second screen with reporter gene assays was applied to the 7 polymorphisms with differential EMSA results. *More single-nucleotide polymorphisms (SNPs) were possible, but they had already been selected in previous groups to the right. RNA-seq = RNA sequencing.

Our aim in the present study has been to identify new functional polymorphisms that could contribute to the protective effect of *IRF5 *haplotypes. Given the location of rs729302 and its linkage disequilibrium (LD) pattern, our focus has been to study polymorphisms 5' to *IRF5 *and in its first intron that could affect transcription through differential binding of nuclear proteins. Our exploration followed an experimental process (Figure 1) initiated by selecting polymorphisms based on tight LD with rs729302 or the protective haplotypes as well as on the basis of their prominent association with *IRF5 *transcription levels. This approach led to the selection of 26 polymorphisms for functional screening. First screening was done by searching allele differential patterns in electrophoretic mobility shift assays (EMSAs) with B cell line nuclear extracts. Seven polymorphisms showed differential binding in these assays and were brought to luciferase reporter gene assays. Three of the seven polymorphisms showed differential luciferase expression with their two alleles. These three new *cis*-regulatory polymorphisms, rs729302, rs13245639 and rs11269962, will allow more accurate understanding of *IRF5 *transcriptional variation and its role in disease susceptibility, as suggested already by our preliminary analysis of their haplotype assignments.

## Materials and methods

### Genotypes, linkage disequilibrium map and haplotype analysis

The region extending from chr7:128,343,695-128,393,203 of the Human March 2006 genome assembly (NCBI36/hg18) that includes the coding sequence of *IRF5*, 21.5 kb of its promoter region (from the start of exon 1, variant A) and part of the transportin 3 (*TNPO3*) locus, which is 3' to *IRF5*, was screened for polymorphisms. Only tag SNPs were selected in about half of this region; in the promoter and first intron of *IRF5*, however, we selected all SNPs characterized by double-hits in NCBI dbSNP Build 128 [18]. The 54 selected SNPs (Additional file 2: Table S1) were analyzed in DNA samples obtained from peripheral blood mononuclear cells (PBMCs) of 95 Spanish healthy controls recruited in our center. They provided their written informed consent to participate in the study, and the study was approved by the Ethics Committee for Clinical Research of Galicia. Genotypes were obtained by single-base extension with the SNaPshot Multiplex Kit (Applied Biosystems, Carlsbad, CA, USA) as described previously [5], except for rs3778752, rs3778751 and the CGGGG indel, which were sequenced; the exon 6 indel, which was genotyped by length variation in agarose gel electrophoresis as described previously [5]; and rs10954213, which was genotyped using a fluorogenic 5' nuclease assay (TaqMan MBG Probes, TaqMan SNP Genotyping Assay; Applied Biosystems). The primers and probes used are given in Additional file 2: Table S2. The genotyping call rate was 97.7%. All the SNPs were in Hardy-Weinberg equilibrium with a threshold for significance of 0.05 without correction for multiple testing. Pairwise D' values, *r*^2 ^values and their graphic representation were obtained using Haploview software at the default settings [19]. Haplotypes were estimated using PHASE 2.1 software (at default parameters), which implements a very accurate Bayesian algorithm [20]. Two haplotype distribution analyses were done, the first to select SNPs for functional studies, including all SNPs of potential interest and the second to put the new functional polymorphisms in context, including only the functional and tag SNPs. Functional screening was conducted on three additional polymorphisms selected on the basis of their relationship with *IRF5 *expression or their known *cis*-regulatory effect. This information is detailed in Additional file 2: Table S3.

### Cell culture and nuclear extracts

The B-lymphocyte cell line WIL2 NS [21,22], which constitutively expresses IRF5 (Additional file 3: Figure S1), was obtained from the European Collection of Cell Cultures (catalogue no. 90112121). It was maintained in RMPI 1640 medium supplemented with 10% fetal bovine serum, 2 mM L-glutamine, 1 U/ml penicillin and 0.1 mg/ml streptomycin. For the preparation of nuclear extracts, 10^7 ^cells were washed in 1 ml of phosphate-buffered saline and incubated at 4°C for 15 min with 200 μl of a lysis buffer containing 10 mM 2-[4-(2-hydroxyethyl)piperazin-1-yl]ethanesulfonic acid (HEPES), pH 7.9, 1 mM ethylenediaminetetraacetic acid (EDTA), 1 mM ethylene glycol tetraacetic acid, 10 mM KCl, 1 mM dithiothreitol (DTT), 1 mM phenylmethylsulfonyl fluoride, 10 μg/ml aprotinin and 10 μg/ml of leupeptin. The freed nuclei were incubated at 4°C for 30 min with 0.1% Triton X-100 and collected by centrifugation at 800 × *g *during 15 min at 4°C. Subsequently, they were incubated at 4°C for 30 min with rotation in 200 μl of the previously specified lysis buffer supplemented with 20% glycerol and 0.4 M KCl. Insoluble material was precipitated by centrifugation for 15 min at 13,000 rpm and 4°C. All the mentioned reagents were obtained from Sigma-Aldrich (St Louis, MO, USA). Nuclear proteins in the supernatant were quantified using the Quant-iT Protein Assay Kit (Molecular Probes, Eugene, OR, USA) in a Qubit fluorometer (Invitrogen, Carlsbad, CA, USA). Quantified nuclear extracts were stored at -80°C until use.

### Electrophoretic mobility shift assay

Oligonucleotides for each allele and strand centered in the 25 selected polymorphisms plus the CGGGG indel used as a positive control were synthesized by Sigma-Genosys (Haverhill, UK) (Additional file 2: Table S4). Complementary oligonucleotides were annealed at 37°C for 1 h to form the double-stranded probes used in EMSA. The LightShift Chemiluminescent EMSA Kit (Pierce Biotechnology, Rockford, IL, USA) was used with some modifications. Briefly, the binding reaction was performed with 0.5 nM of double-stranded, biotin-labeled oligonucleotides and 0.5 to 4.0 μg of nuclear extract proteins from WIL2 NS cells. This reaction was brought to a final volume of 20 μl with binding buffer (10 mM Tris·HCl, pH 7.5, 10 mM KCl, 0.6 mM DTT, 50 ng/μl poly(deoxyinosinic-deoxycytidylic) acid, 1.5 mM EDTA, 2 mM HEPES, pH 7.9, 25 mM NaCl, 0.1 mM ZnSO_4_, 15% glycerol and 0.25 mg/ml bovine serum albumin) and incubated at room temperature for 20 min. Cold competition was done with a 200-fold molar excess (0.1 μM) of the unlabeled oligonucleotide. DNA-protein complexes were analyzed by electrophoresis in 5% polyacrylamide nondenaturing gels and 0.5 × Tris/borate/EDTA buffer at 11 V/cm and 4°C for 3 h. Subsequently, they were transferred to nylon membranes according to a semidry transfer protocol for 45 min at 12 V. The complexes were cross-linked to the nylon membrane with ultraviolet (UV) light at 7.2 J/cm^2^. Detection was done with the LightShift Chemiluminescent EMSA Detection Module (Pierce Biotechnology) followed by exposure to UV light using the UVP EC3 Imaging System (UVP, Upland, CA, USA). A minimum of three independent replicate experiments were done for each EMSA showing differential binding.

### Luciferase reporter assays

Fourteen different constructs were made, one for each of the alleles of the seven polymorphisms with differential EMSA results. The *IRF5 *sequence (148 to 245 bp in length) of each of these polymorphisms was obtained from heterozygous subjects by polymerase chain reaction (PCR) with the same primers used for genotyping (Additional file 2: Table S5). None of them included additional polymorphisms. The amplicons were first inserted into the EcoRV site of a pBlueScript (pBK SK-) vector by TA cloning. Briefly, the EcoRV cut vector was treated with Taq DNA polymerase in the presence of 2 mM deoxythymidine triphosphate and PCR buffer, and 50 μg of this 3' T-vector were ligated to 25 μg of the PCR amplicon in a 10-μl reaction with 1 μl of T4 DNA ligase reaction buffer (New England Biolabs, Ipswich, MA, USA) and 1× ligase buffer at 16°C overnight. The cloned *IRF5 *sequences were subcloned into the KpnI/SmaI sites of a *fos*-pGL3-basic vector, which are just upstream of the minimal *fos *promoter regulating expression of the *Firefly *luciferase gene. The sequence and orientation of each insert were verified by DNA sequencing. These verified reporter vectors were transfected in WIL2 NS cells. Specifically, each experiment included five conditions: blank, positive control, empty vector, allele 1 and allele 2. The experiments were carried out by transfecting 2 × 10^6 ^WIL2 NS cells with 400 ng of the adenovirus E1 region RSV-Luc vector plus 2,800 ng of the pBK SK- vector for the positive control, 3,200 ng of the *fos*-pGL3 basic vector for the empty vector, 3,200 ng of the allele 1 *fos*-pGL3 vector construct, and 3,200 ng of the allele 2 *fos*-pGL3 vector construct for each of the two alleles. All transfections also included 800 ng of the pRL-TK *Renilla *luciferase vector (Promega, Madison, WI, USA) for normalization. Transfections were done by microporation in 100 μl of Resuspension Buffer R at 1,100 V for 30 ms and a single-pulse program in the Neon Transfection System (Invitrogen). After microporation, the transfected cells were left in culture for 24 h (basal) or left for 20 h. The luciferase activity was measured using the *Firefly *Luciferase and the *Renilla *Luciferase Assay systems (both from Promega). The results for *Firefly *luciferase were expressed as relative luciferase units after normalization with the *Renilla *luciferase activity and with the empty vector for the respective experiments. At least five independent experiments were performed for each of the seven analyzed polymorphisms. Comparison of the log-transformed mean normalized *Firefly *luciferase luminescence ratios was done using the Wilcoxon matched-pairs test.

## Results

### Selection of promoter polymorphisms on the basis of linkage disequilibrium and haplotypes

The experimental design involved selection of SNPs on the basis of three criteria (Figure 1): high *r*^2 ^values with rs729302, high correlation with the protective haplotypes and prominent association with *IRF5 *expression. These three criteria are not mutually exclusive, and many SNPs were selected for more than one of them. The selected SNPs were evaluated for their effect in binding nuclear proteins as an indication of their possible role in *cis*-regulation of *IRF5 *expression by means of EMSA. The SNPs with differential binding to their alleles in EMSA were assessed with reporter gene assays, and those showing changes in expression between the constructs with the two alleles were considered to be functional (Figure 1).

**Figure 2:**
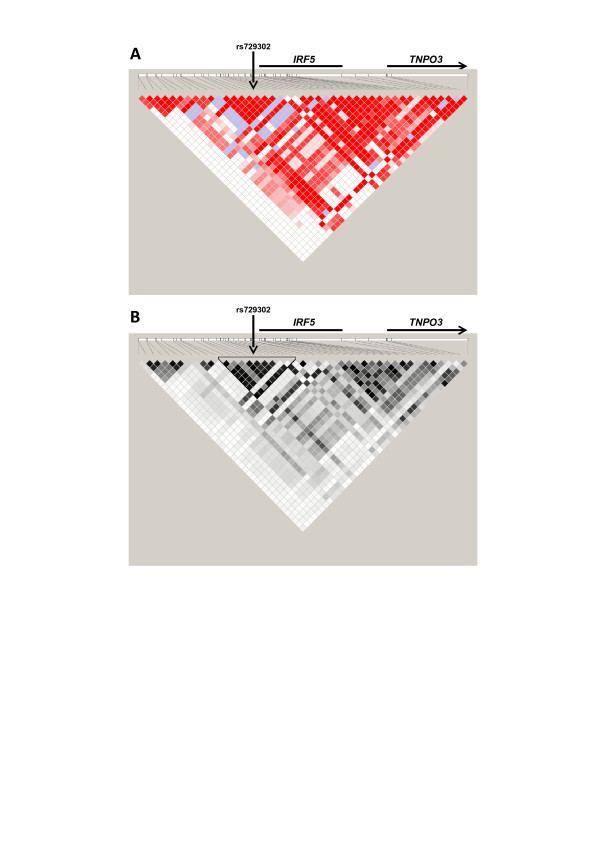
***IRF5 *linkage disequilibrium maps**. Pairwise D' **(A) **and *r*^2 ^**(B) **between 44 polymorphisms in the *IRF5 *locus obtained in 95 Spanish healthy controls are represented. Ten additional single-nucleotide polymorphisms (SNPs) were excluded because of low minor allele frequency (<2%) or complete redundancy with other SNPs (see Results). Color scale from white to red indicates D' values from 0 to 1 in (A) and that from white to black indicates *r*^2 ^values from 0 to 1 in (B). The triangle in (B) surrounds the block containing SNPs in tight linkage disequilibrium with rs729302. Positions of the coding sequences of *IRF5 *and *TNPO3 *are signaled.

**Figure 3:**
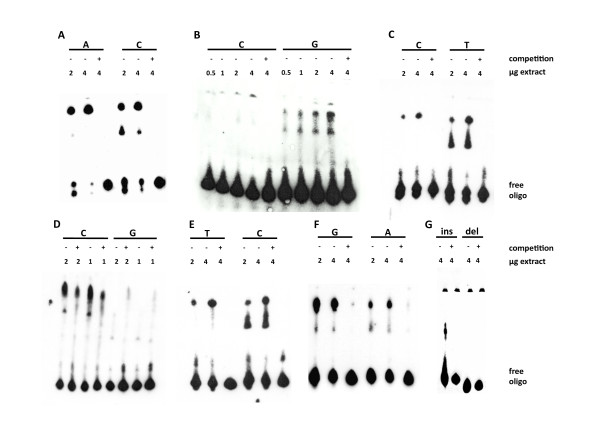
**Seven *IRF5 *polymorphisms showing differential electrophoretic mobility shift assay results**. Seven of the twenty-six polymorphisms analyzed by electrophoretic mobility shift assay (EMSA) showed differential patterns of delayed migration with their two alleles. **(A) **rs729302. **(B) **rs12706860. **(C) **rs13245639. **(D) **rs3778754. **(E) **rs3807307. **(F) **rs17424179. **(G) **rs11269962. They were analyzed with WIL2 NS cell nuclear extracts, and the specificity of the bands was checked by competition with unlabeled oligonucleotides in 200-fold excess. Each difference was established in three independent EMSAs with concordant results. Sequences of the probes are given in Additional file 2: Table S4.

To identify SNPs strongly correlated with rs729302 or with the protective haplotypes, we genotyped 54 polymorphisms located predominantly within 21.5 kb of the 5' region of *IRF5 *(Additional file 2: Table S1) in 95 Spanish controls. The D' and *r*^2 ^pairwise values were calculated for 44 polymorphisms (Additional file 2: Table S6), which excluded eight SNPs with minor allele frequencies less than 2% (rs12536195, rs6951615, rs11773414, rs11983607, rs11763323, rs754280, rs41298401 and rs11767834) and two SNPs that were completely redundant with rs3778753 (rs3778752 and rs3778751). This analysis showed a block of LD extending over 43 kb from *TPNO3 *to the 5' region of *IRF5 *further from the gene than rs729302 (Figure 2A). This block included 37 of the analyzed SNPs. A second block of LD with seven SNPs was even further 5' to *IRF5 *than the first. In the largest block of LD, there were two areas of stronger correlation between SNPs (Figure 3B), one of them (8 kb) included rs729302 and other eight SNPs (*r*^2 ^≥ 0.7 with rs729302). These nine SNPs were selected for functional screening (Table 1). The pattern of LD was very similar in the 379 samples of European ancestry in phase 1 of the 1000 Genomes project (Additional file 3: Figure S2) [23].

**Table 1:** Polymorphisms in the *IRF5 *locus screened by functional tests.^a^

Polymorphism	Position	Gene	Alleles	MAF	Selection criteria^b^	EMSA	Reporter
rs4731530	128562578	5' *IRF5*	C > T	39.9	Hap	-	
rs6950728	128565661	5' *IRF5*	G > A	30.9	*r*^2 ^= 0.97	-	
rs11982901	128566754	5' *IRF5*	C > T	31.6	*r*^2 ^= 0.97	-	
rs4728141	128567032	5' *IRF5*	C > T	49.5	Hap	-	
rs13245639	128567810	5' *IRF5*	C > T	31.6	*r*^2 ^= 0.97	+	+
rs729302	128568960	5' *IRF5*	A > C	31.1	*r*^2 ^= 1.0	-	
rs729068	128569561	5' *IRF5*	C > T	31.1	*r*^2 ^= 0.9	-	
rs12706860	128570026	5' *IRF5*	C > G	33.7	*r*^2 ^= 0.83	+	+
rs7808659	128570238	5' *IRF5*	C > A	30.4	*r*^2 ^= 0.9	-	
rs754284	128571478	5' *IRF5*	C > G	31.6	*r*^2 ^= 0.87	-	
rs4728142	128573967	5' *IRF5*	G > A	47.8	Hap	-	
rs7801838	128573994	5' *IRF5*	C > T	28.9	*r*^2 ^= 0.73	-	
rs3778754	128575552	5' *IRF5*	G > C	48.9	Hap	+	-
rs11269962	128575812	5' *IRF5*	ins > del	48.9	RNA-seq	+	+
rs3757388	128576023	5' *IRF5*	A > G	33.3	Hap	-	
rs3757387	128576086	5' *IRF5*	T > C	49.4	Hap	-	
rs3757385	128577304	5' *IRF5*	G > T	33.3	Hap	-	
rs3807135	128577617	5' *IRF5*	C > T	33.0	Hap	-	
CGGGG indel	128577931	5' *IRF5*	del > ins	48.4	Known *cis*-eQTL	+	+
rs3807307	128579202	*IRF5*	C > T	47.8	Hap	+	-
rs3823536	128579666	*IRF5*	A > G	47.8	Hap	-	
rs3778753	128580042	*IRF5*	G > A	47.8	Hap	-	
rs3778752	128580047	*IRF5*	T > G	47.8	Hap	-	
rs3778751	128580048	*IRF5*	T > A	47.8	Hap	-	
rs3807306	128580680	*IRF5*	T > G	45.7	Hap/microarray	-	
rs17424179	128657995	*TPNO3*	G > A	3.8^c^	Microarray	+	-

^a^The polymorphisms' genomic position, relation to genes, alleles, minor allele frequency (MAF), criteria of selection and results of the electrophoretic mobility shift assay (EMSA) and reporter gene assays are shown. ^b^Selection criteria were hap = concordance of the minor allele with the minor allele of rs729302 in most haplotypes; *r*^2 ^= level of genotype correlation with rs729302; RNA-seq = polymorphism most associated with RNA-seq data; microarray = specially associated single-nucleotide polymorphisms in microarray expression analysis. ^c^Frequency from HapMap (Hap) CEPH (Utah residents with ancestry from northern and western Europe: CEU) individuals. eQTL = expression quantitative trait loci.

Next, we studied the haplotypes of the 37 SNPs in the large LD block. There were 20 haplotypes with frequencies over 1% that represented 85.3% of the chromosomes in the studied samples (Table 2). Thirteen of the twenty common haplotypes could be traced back as subhaplotypes of the six haplotypes defined with tag SNPs in our previous studies (and described in Additional file 1: Note S1) [5,12,15]. The other seven haplotypes were new. On the basis of these data, we selected for functional analysis the 15 SNPs with minor alleles in the haplotypes that generally (>50%) carried the minor allele of rs729302 (Table 2).

**Table 2:** Haplotypes of *IRF5 *used to select polymorphisms.^a^

Haplotype^b^	%	rs4731530	rs2402941	rs6968225	rs6968508	rs4728141	*rs729302*	rs2402940	rs754281	rs11768806	rs4728142	rs1874330	rs3778754	rs3757388	rs3757387	rs3757385	rs3807135	*rs2004640*	rs3807307	*rs752637*	rs3823536	rs3778753 ^c^	rs3807306	EXON 6 INDEL	rs10954213	*rs13242262*	*rs10488630*	*rs10488631*	*rs2280714*	*rs4731535*
4	24.7	C	G	C	C	C	*A*	C	C	C	A	T	G	A	C	G	C	*T*	C	C	A	G	T	A	A	*T*	*G*	*T*	*T*	*T*
4	1.1	-	-	-	-	-	-	-	-	-	**-**	-	-	-	-	-	-	-	-	-	-	-	G	-	-	-	-	-	-	-
4	3.2	T	-	-	-	-	-	-	-	-	-	-	-	-	-	-	-	-	-	-	-	-	-	-	-	-	-	-	-	-
na	1.6	-	-	-	-	-	-	-	-	-	-	-	-	-	-	-	-	-	-	T	-	-	-	-	-	-	-	-	-	-
5	3.2	-	-	-	-	-	-	-	-	-	-	-	-	-	-	-	-	-	-	-	-	-	-	-	-	-	*A*	-	-	-
6	6.8	-	-	-	-	-	-	-	-	-	-	-	-	-	-	-	-	-	-	-	-	-	-	G	-	-	*A*	*C*	-	*C*
6	2.6	-	-	-	-	-	-	-	-	-	G	-	-	-	-	-	-	-	-	-	-	-	-	G	-	-	*A*	*C*	-	*C*
na	1.6	-	-	-	-	T	-	-	-	-	G	-	-	-	T	-	-	-	-	T	-	-	-	-	-	-	*A*	-	-	*-*
na	1.6	**T**	-	-	-	**T**	*C*	-	-	-	-	-	-	-	-	-	-	-	-	-	-	-	-	-	-	-	*-*	-	-	*-*
na	2.6	-	-	-	-	T	-	-	-	-	G	-	C	-	T	-	-	-	T	-	G	A	G	G	G	*A*	*A*	-	-	*C*
na	1.1	-	-	-	-	T	-	-	-	-	G	-	C	-	T	-	-	-	T	T	G	A	G	G	G	*A*	*A*	-	-	*C*
3	7.9	T	A	G	G	T	-	T	T	T	G	C	C	G	T	T	T	*G*	T	T	G	A	G	G	G	*A*	*A*	-	*C*	*C*
3	1.1	T	A	G	G	T	-	T	-	-	G	-	C	G	T	T	T	*G*	T	T	G	A	G	G	G	*A*	*A*	-	*C*	*C*
3	2.6	T	-	-	-	-	-	T	T	T	G	C	C	G	T	T	T	*G*	T	T	G	A	G	G	G	*A*	*A*	-	*C*	*C*
2	7.9	**T**	-	-	-	**T**	*C*	-	-	-	**G**	-	**C**	**G**	**T**	**T**	**T**	*G*	**T**	T	**G**	**A**	**G**	G	G	*A*	*A*	-	*C*	*C*
2	1.1	**T**	-	-	-	**T**	*C*	-	-	-	**G**	-	**C**	**G**	**T**	**T**	**T**	*G*	**T**	T	**G**	**A**	**G**	G	G	*A*	*A*	-	*C*	*C*
2	3.2	-	-	-	-	**T**	*C*	-	-	-	**G**	-	**C**	**G**	**T**	**T**	**T**	*G*	**T**	T	**G**	**A**	**G**	G	G	*A*	*A*	-	*C*	*C*
1	5.3	**T**	-	-	-	**T**	*C*	-	-	-	**G**	-	**C**	-	**T**	-	-	*G*	**T**	-	**G**	**A**	**G**	**-**	**-**	-	*A*	-	-	-
na	3.2	-	-	-	-	**T**	*C*	-	-	-	**G**	-	**C**	-	**T**	-	-	*G*	**T**	-	**G**	**A**	**G**	**-**	**-**	-	-	-	-	-
na	3.2	**T**	-	-	-	**T**	*C*	-	-	-	**G**	-	**C**	**G**	**T**	**T**	**T**	*G*	**T**	T	**G**	**A**	**-**	**-**	**-**	-	-	-	-	-

^a^Dashes represent the same allele as the uppermost sequence. Minor alleles >50% concordant with the C allele of rs729302 are in bold, and the name of the polymorphism is also in bold. *IRF5 *tag single-nucleotide polymorphisms (SNPs) are in italics. The eight SNPs with high correlation with rs729302 are not represented. ^b^Haplotype numbers are as previously described. na = not applicable for lack of correspondence. ^c^Three completely redundant SNPs

### Selection of polymorphisms on the basis of their association with *IRF5 *expression

A recent analysis by our group, in which we combined data from multiple microarray experiments in lymphoblastoid cell lines, highlighted four *IRF5 *polymorphisms in the best models accounting for *IRF5 *expression [12]. We selected three of them for the current study. One of them is the CGGGG indel, which is already known to influence *IRF5 *expression [10,11]. It was selected as a sort of positive control (Table 1). The second is rs3807306, which had already been selected on the basis of our haplotype analysis. The third is rs17424179, which was not included in our LD analysis because it is 68 kb 3' to *IRF5 *and we selected it at this stage. The fourth SNP in the microarray-derived model, rs10954213, was not selected because it is in the *IRF5 *coding sequence and affects mRNA stability.

We selected an additional polymorphism on the basis of *IRF5 *expression data obtained using the RNA-seq approach (E Alonso-Perez, unpublished data). More details are available in Additional file 1: Note S2. Briefly, the transcriptome of the 60 cell lines of the HapMap CEPH (Utah residents with ancestry from northern and western Europe: CEU) collection was obtained from a published study [24]. After alignment of the RNA-derived sequences, reads corresponding to exons were counted together with the junction reads, and the total was normalized and used as a measure of the expression level. This measure for each cell line was used as the variable to explain the genotypes of 264 *IRF5 *polymorphisms from the 1000 Genomes project. The most significantly associated polymorphism in this analysis was rs11269962 (*P *= 0.002), which is a 14-bp indel placed 2.2 kb upstream of the *IRF5 *gene (Table 1).

### Screening of functional polymorphisms with electrophoretic mobility shift assay

We considered it likely that the SNPs of interest could have a functional effect dependent on the differential allele binding of nuclear proteins. EMSA was used as a screening tool for these differences. We used nuclear extracts from a lymphoblastoid cell line in basal conditions. A total of 26 polymorphisms were subjected to this screening (Figure 1): 9 in the rs729302 high-correlation group, 15 from the haplotype selection and 2 on the basis of their association with *IRF5 *expression (Table 1). In addition, we analyzed the CGGGG indel as a positive control (Additional file 3: Figure S3A).

Only 7 of the 26 polymorphism showed differential allele patterns in EMSA. The first was rs729302 itself, which showed additional binding with the C allele relative to the A allele (Figure 3A). Two others were from the group in tight LD with rs729302. The minor G allele of rs12706860 showed a specific pattern of additional bands with delayed migration when it was compared with the major C allele (Figure 3B). The other SNP of this group, rs13245639, showed specific band slowdown with its minor T allele in comparison with the major C allele (Figure 3C). Also, two of the SNPs selected on the basis of *IRF5 *protective haplotypes, rs3778754 and rs3807307, showed differential EMSA results. The G allele of rs3778754 showed delayed bands that were much more prominent with the C allele than with the G allele (Figure 3D). Interpretation of this difference was complicated by the lack of specificity of the G allele band, which was attenuated by competition with the unlabelled oligonucleotide but not completely suppressed. In spite of this lack of specificity, other differences between EMSAs with the two alleles led us to select this SNP for further study. The next SNP, rs3807307, showed an additional and specific band shift with the major C allele that migrated faster than the band that was common to the two alleles (Figure 3E). The two polymorphisms selected on the basis of their prominent association with *IRF5 *expression also showed differential EMSA results. For rs17424179, there were two shifted bands with the two alleles; however, the slowest band was much more intense than the fastest one with the G allele, and this difference was not as marked for the A allele (Figure 3F). For the major allele of rs11269962, the 14-bp insertion, a specific delayed band was observed that was absent with its minor allele (Figure 3G).

These differential patterns of binding with nuclear extracts of the B cell line WIL2 NS were obtained in three or more concordant experiments. None of the other 19 analyzed polymorphisms showed differential results in EMSA. Among them was rs4728142, which has been reported to show differential allele intensity of the delayed band [25]. We analyzed this SNP with particular attention, but we did not detect any difference between the two alleles (Additional file 3: Figure S4).

### Luciferase gene reporter assays

To further assess the functionality of the seven polymorphisms that had shown differential EMSA results (Table 1), we performed luciferase gene reporter assays. Each polymorphism was tested with a pair of vectors: one for each allele (148 to 245 bp in length; see Additional file 2: Table S5). They did not contain any other SNP. Inserts for the seven polymorphisms plus those used as positive controls (CGGGG indel) were introduced upstream of the *fos *minimal promoter in *fos*-pGL3 vectors. These constructs were transiently transfected into the B-lymphocyte cell line WIL2 NS. The CGGGG indel showed higher expression with the 4x allele than with the 3x allele (Wilcoxon test; *P *= 0.043), as described previously [11] (Additional file 3: Figure S3B).

**Figure 4:**
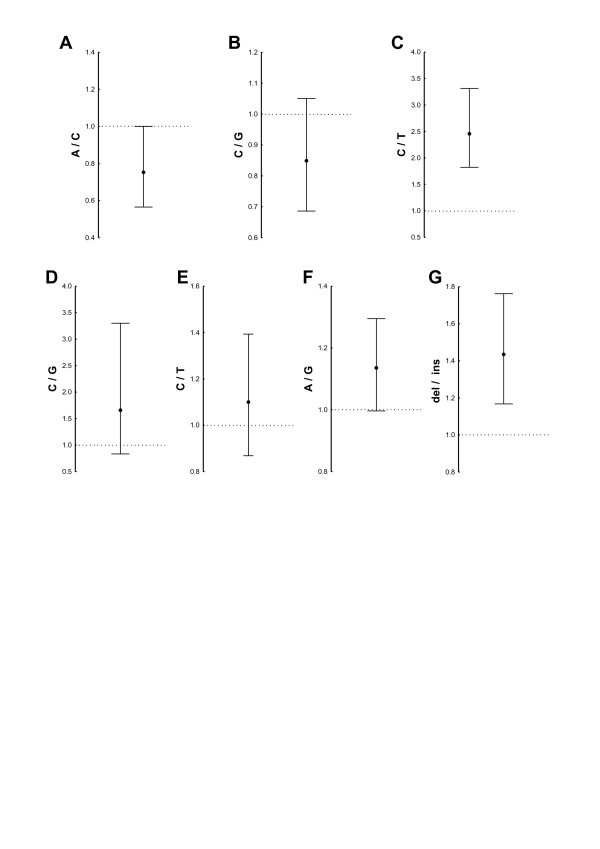
**Reporter gene assays of the seven *IRF5 *polymorphisms with differential electrophoretic mobility shift assays**. Each graph represents the geometric mean and the 95% confidence interval of ratios between the luciferase signals of the WIL2 NS cells transfected with the two alleles of **(A) **rs729302, **(B) **rs12706860, **(C) **rs13245639, **(D) **rs3778754, **(E) **rs3707307, **(F) **rs17424179 and **(G) **rs11269962. The dotted horizontal line corresponds to a ratio of 1.0 or identical expression with the two alleles. *P < 0.05 by Wilcoxon matched-pairs test of five independent experiments, except for rs12706860, for which seven experiments were done.

Three of the seven newly studied polymorphisms showed differential luciferase expression: (1) rs729302 itself (Figure 4A), (2) rs13245639 (Figure 4C), both from the group of SNPs in tight LD with rs729302, and (3) the 14-bp indel rs11269962 (Figure 4G). The minor C allele of rs729302 showed increased expression relative to the major A allele, but the difference was only borderline (Figure 4A). Constructs with the major C allele of rs13245639 showed a significant increase in luciferase expression compared with the minor T allele (Figure 4C). The construct with the 14-bp indel of rs11269962 showed a significant decrease in luciferase expression relative to the construct with the deletion allele (Figure 4G). These latter results were consistent with the RNA-seq analysis, which showed higher expression with the deletion allele. The remaining four SNPs with differential EMSA results did not show significant differences in the reporter gene assays (Figures 4B, D, E and 4F).

### Haplotype assignments of the new functional polymorphisms

We performed an estimation of haplotypes to facilitate interpretation of the newly identified functional polymorphisms. The three new functional polymorphisms, comprising the two known *cis*-regulatory polymorphisms (CGGGG indel and rs10954213) and the eight tag SNPs used in our previous studies (total of thirteen polymorphisms), were included in this analysis. In this way, it was possible to explore retrospectively correspondences between the functional polymorphisms and *IRF5 *mRNA expression or association with SLE, as observed in previous studies [5,12]. We obtained nine haplotypes of relative high frequency that accounted for 83.2% of the total (Table 3). They included the six previously studied (described in Additional file 1: Note S1) and three new ones, which were excluded from further analysis because of lack of information. The remaining six haplotypes still accounted for 74.2% of the total, allowing for meaningful analysis. We computed a sum score of the alleles of the five functional polymorphisms assigned to each haplotype in function of the increased (H) or decreased (L) regulation associated with them (Table 3). These sum scores were correlated with the corresponding *IRF5 *expression or association with SLE. For expression, we used the weighted means of the haplotype-specific regression coefficients in four microarray experiments with lymphoblastoid cell lines [12]. Correlation between the expression levels and the sum score of the *cis*-regulatory polymorphisms was very good (*r*_s _= 0.93, *P *= 0.008). In contrast, correlation of these sum scores with the effect size (odds ratio) of each haplotype in SLE association [5] was high but not significant (*r*_s _= 0.74, *P *= 0.09).

**Table 3:** Assignment of *cis*-regulatory polymorphisms to haplotypes with associated mRNA expression and systemic lupus erythematosus susceptibility.^a^

Haplotype^b^	%	Alleles at haplotype^c^	Allele effect^d^	Expression	Association
1	5.3	TCIDGCATATTT	LHLLH	0.24 (-0.3 to 0.8)	0.75 (0.6 to 0.9)
2	14.2	TCIDGTGAATCC	LHLLL	-1.10 (-1.5 to -0.7)	0.79 (0.7 to 0.9)
3	13.7	CAIDGTGAATCC	HLLLL	-1.33 (-1.7 to -1.0)	0.89 (0.8 to 1.1)
4	29.5	CADITCATGTTT	HLHHH	0.84 (0.5 to 1.2)	0.95 (0.8 to 1.1)
5	2.6	CADITCATATTT	HLHHH	1.63 (0.7 to 2.6)	1.06 (0.8 to 1.4)
6	8.9	CADITCATACTC	HLHHH	1.38 (1.1 to 1.7)	1.93 (1.6 to 2.3)
na	3.7	TCIDGCATGTTT			
na	2.6	CAIDTCGAATTC			
na	2.6	TCIDGTATGTTT			

^a^Five *cis*-regulatory polymorphisms and seven tag SNPs are represented. Allele effects to increased (H) or decreased (L) expression are signaled. Combinations of these effects were retrospectively correlated with the mean specific expression in unstimulated lymphoblastoid cells (95% C.I.)[12] and OR (95% C.I.) of the association with SLE[5]. ^b^Haplotype numbers as previously described. na = not applicable for lack of correspondence. ^c^Order of polymorphisms is: rs13245639, rs729302, rs11269962, CGGGG indel, rs2004640, rs752637, rs10954213, rs13242262, rs10488630, rs10488631, rs2280714 and rs4731535. ^d^Order of allele effects: rs13245639, rs729302, rs11269962, CGGGG indel and rs10954213.

## Discussion

We have identified three new functional polymorphisms in the 5' region of *IRF5 *that contribute to the complex scenario of *cis*-regulation of this gene. These polymorphisms have been identified by concordant results in EMSA and gene reporter assays with a B cell line, which is a relevant cell type for SLE pathogenesis. Therefore, it is likely that the three polymorphisms affect *IRF5 *levels by the differential binding of transcription factors or other regulatory proteins. Their identification opens new possibilities for research to obtain a more accurate understanding of *IRF5 *transcriptional variation between subjects, as well as the identification of transcription factors with critical *in vivo *roles. In addition, this type of knowledge will allow refined understanding of the participation of *IRF5 *in disease susceptibility. First evidence of the future possibilities was obtained with the retrospective analysis of the assigned haplotype associations with *IRF5 *expression and SLE susceptibility.

Identification of the three new functional polymorphisms was based on differences in EMSA and reporter gene assays. All these assays have been replicated, and therefore we are confident in the results. However, these assays and their interpretation can be problematic in some situations. For example, there were two EMSAs with difficult interpretation: for rs3778754 due to lack of specificity of a band and for rs1742417 because only differences in intensity were found. In these two cases, reporter gene assays were negative, allowing us to concentrate on the most consistent results. Further confidence in the positive findings could be gained from studying additional cell lines. We did not check other cells, but one of the newly identified functional SNPs, the 14-bp indel rs11269962, is in a region identified as a strong enhancer in another lymphoblastoid cell line (GM12878) by the Encyclopedia of DNA Elements (ENCODE) Consortium's ENCODE project [26], which has analyzed the whole genome for regulatory sequences.

We used sensitive techniques, both in EMSA and in the reporter gene assay. In this respect, the use of a weak promoter in the reporter gene assays, as well as the use of normalization of transfection efficiency with the cotransfected *Renilla *luciferase signal, is of particular relevance. In addition to these general procedures, we analyzed EMSA for rs4728142 with special attention, given the previously reported differences [25], but we found no differences. Our results do not exclude other *IRF5 cis*-regulatory polymorphisms in response to stimuli or those specific to other cell types, but cell-specific regulatory polymorphisms are a small minority [27,28]. Also, our study did not address specific effects of *IRF5 *alternative splicing isoforms and avoided expression data from exons with multiple isoforms, such as exon 1. In addition, it did not address many other polymorphisms in the locus. In addition, the known differences in *IRF5 *haplotypes, as well as their SLE association and *cis*-regulation between Europeans, Asians and Africans [29-32], do not allow extension of our findings to other ethnic groups without studying them (Additional file 1: Note S1). In spite of this caution, we noted that the functional SNP rs13245639 showed *r*^2 ^values over 0.94 with rs729302 in East Asian and African samples from phase 1 of the 1000 Genomes project [23]. This result suggests that rs13245639 could play a similar role in SLE susceptibility in these ethnic groups. In contrast, the third functional SNP, rs11269962, showed *r*^2 ^= 0.36 with rs729302 in our samples and below 0.05 in both Asians and Africans.

The three new *cis*-regulatory polymorphisms complete the two that were already known. This number of *cis*-regulatory polymorphisms is large but not surprising. A high number is consistent with the findings of experiments looking for *IRF5 *expression quantitative trait loci in lymphoblastoid cell lines [33-36]. These studies found that most SNPs in *IRF5 *were associated with its mRNA levels. This result cannot be explained with one or two *cis*-regulatory SNPs. Also, previous analyses have shown that the best models accounting for variation in *IRF5 *expression require combinations of several *cis*-regulatory SNPs [12]. In addition, multiple functional *cis*-regulatory polymorphisms in the same gene have already been found in loci associated with other complex diseases [37-39]. The complex structure of gene regulation that has been uncovered in recent years, involving multiple molecular interactions, specifically the binding with DNA of multiple transcription factors [40], makes it likely that this type of finding will become more common with progress in the identification of causal polymorphisms.

A limitation of our study is that it did not assess the disease significance of the findings in patients and controls. However, it should be noted that this type of analysis has many difficulties. The number of *cis*-regulatory polymorphisms and the presence of LD between them makes it very difficult to discern their specific contributions in epidemiological or *ex vivo *studies. Only a small fraction of the many possible combinations of *cis*-regulatory polymorphisms are present in the population at a significant frequency. Therefore, the observable expression levels do not allow the distinction of individual effects of each polymorphism. In this way, the *in vivo *relevance of the *cis*-regulatory polymorphisms is only inferred, because it is very difficult to replicate the results demonstrated in *in vitro *analysis. These difficulties have already been encountered in previous studies that failed to replicate the *IRF5 in vitro *results in studies done in PBMCs from SLE patients and healthy controls [13,41]. These analyses will be much more difficult to perform with the addition of the three functional SNPs described herein that show LD between them and with the CGGGG indel (as shown in the haplotype analysis in Table 3). An alternative will be to demonstrate that the polymorphisms are in transcription factor-binding sites (TFBSs) that are functional *in vivo *and that these TFBSs are disrupted by one of the two alleles. These experiments can be done *ex vivo *with cells taken from patients or healthy controls using techniques such as chromatin immunoprecipitation [42]. To do these analyses, we need to identify the relevant transcription factors. Our repeated attempts were unsuccessful (data not shown). They were based on supershift assays with specific antibodies against the most likely transcription factors according to the known TFBSs. These negative results are not surprising, because detection of TFBSs with available bioinformatics tools is notoriously inefficient [43]. However, we plan to pursue the search by taking advantage of a new approach based on proteomic analysis [39] that is more accurate, although very complex to set up.

The phenotypes in cells or in patients are the sum of the functional alleles present. Therefore, the relevant units of ascertainment of the expression levels are the haplotypes containing the various functional polymorphisms, which we have applied in our current study. For this analysis, we took advantage of previously defined haplotypes and their association with *IRF5 *expression and with SLE susceptibility. Correlation of the sum scores of *cis*-regulatory polymorphisms in each haplotype with *IRF5 *expression in lymphoblastoid cells was very good. This result is only preliminary, but it is promising. We expect that direct assessment of haplotypes defined by the functional polymorphisms will be even more correlated with the expression levels. It is also likely that the modest correlation of the sum scores observed in our current study will improve in case-control association studies that include all the functional polymorphisms. However, it has already been signaled that SLE's association with *IRF5 *is unlikely to be fully explained by *cis*-regulatory polymorphisms [12,41]. Other changes in *IRF5 *function beyond mRNA expression levels seem to be needed.

## Conclusions

We have identified three new functional *cis*-regulatory *IRF5 *polymorphisms. Together with the two that were already known, they correlate very well with previously defined haplotype effects on *IRF5 *expression. Knowledge of these *IRF5 *functional polymorphisms will increase the power of association and expression quantitative trait loci studies because power is always greater for causal polymorphisms than for markers in imperfect LD with them. They will also increase our capacity to understand the role of *IRF5 *in disease pathogenesis.

## Abbreviations

CEU: CEPH (Utah residents with ancestry from northern and western Europe)from the International HapMap Project; EMSA: Electrophoretic mobility shift assay; *IRF5*: Interferon regulatory factor 5; LD: Linkage disequilibrium; MAF: Minor allele frequency; OR: Odds ratio; PBMC: Peripheral blood mononuclear cell; PCR: Polymerase chain reaction; RNA-seq: RNA sequencing; *r*_s_: Spearman rank-order coefficient; Sp1: Specificity protein 1; TFBS: Transcription factor binding site; *TNPO3*: Transportin 3

## Competing interests

The authors declare that they have no competing interests.

## Authors' contributions

EAP performed the EMSA and gene reporter experiments and also participated in interpretation of the results and writing the manuscript. RFP participated in the EMSA and gene reporter experiments. MC participated in analysis of the results. EL, TK and JM provided the RNA-seq data and participated in data analysis and interpretation. JJGR participated in the analysis of the results. AG designed the study, supervised its execution and participated in writing the manuscript. All authors participated in interpretation of the data and results, revised the manuscript with important intellectual content and read and approved the final manuscript.

## Supplementary Material

Additional file 1 **Note S1**: ***IRF5 *haplotypes defined in previous studies and their relationship with systemic lupus erythematosus susceptibility and *IRF5 *expression**. Note S2: RNA-seq study of the *IRF5 *transcriptome and detection of potential *cis*-regulatory polymorphisms. Click here for file

Additional file 2 **Table S1 Polymorphisms included in our analysis with indication of their genomic position, relationship with known genes, alleles and minor allele frequency (MAF) and whether they were present in HapMap and in the 1000 Genomes database**. Table S2 Oligonucleotides used for genotyping. Table S3: Three polymorphisms included in the study for their association with *IRF5 *expression level or known *cis*-regulatory effect. Table S4: Polymorphisms analyzed by electrophoretic mobility shift assay with indication of the oligonucleotides used as probes. Table S5: Primers used to generate the inserts included in the plasmid vectors for reporter gene assays. Table S6: Pairwise linkage disequilibrium relationship between the analyzed polymorphisms. Click here for file

Additional file 3 **Figure S1 Western blot showing expression of *IRF5 *in WIL2 NS cells**. Figure S2 Relationship of the *r*^2 ^values between rs729302 and the other single-nucleotide polymorphisms included in our study obtained in our samples and in phase 1 of the 1000 Genomes projec. Figure S3 Functional analysis of the positive control: the CGGGG indel. Figure S4 Lack of difference between the two alleles of rs4728142 in electrophoretic mobility shift assay. Click here for file
