# Supplementary material for: Identification of three new cis-regulatory IRF5 polymorphisms: in vitro studies
Source: Arthritis Res Ther. 2013 Aug 13;15(4):R82. doi: 10.1186/ar4262 (PMC3978921; doi:10.1186/ar4262)
Supplement: Additional file 1 — Note S1: IRF5 haplotypes defined in previous studies and their relationship with systemic lupus erythematosus susceptibility and IRF5 expression. Note S2: RNA-seq study of the IRF5 transcriptome and detection of potential cis-regulatory polymorphisms. [file ar4262-S1.DOC]

**Supplementary Note 1**

***IRF5* haplotypes defined in previous studies and their relationship with SLE susceptibility and *IRF5* expression**

Using eight IRF5 tagSNPs we defined with Phase 2.1 software six haplotypes with frequency over 5 % in an association study with 1383 SLE patients and 1614 controls all of them of European ancestry[1]. Haplotypes #1 and 2 were associated with lower SLE risk, haplotypes #3, 4 and 5 were neutral and haplotype #6 was associated with increased risk as shown in the following table (modified from reference 1).

| # | Haplotype^a^ | % in Controls | % in SLE | O.R. (95 % C.I.) |
| --- | --- | --- | --- | --- |
| 1 | CGGTATAC | 7.7 | 5.9 | 0.75 (0.6-0.9) |
| 2 | CGAAATGT | 14.3 | 11.6 | 0.79 (0.7-0.9) |
| 3 | AGAAATGT | 14.0 | 12.7 | 0.89 (0.8-1.1) |
| 4 | ATGTGTAC | 31.3 | 30.1 | 0.95 (0.8-1.1) |
| 5 | ATGTATAC | 5.2 | 5.4 | 1.06 (0.8-1.4) |
| 6 | ATGTACAT | 10.0 | 17.7 | 1.93 (1.6-2.3) |

^a^ SNP order was rs729302, rs2004640, rs752637, rs13242262, rs10488630, rs10488631, rs2280714 and rs4731535.

Further analyses showed that alleles of none of the functional polymorphisms known until now matched with either the risk or the protective haplotypes: the minor allele of rs2004640 creating a splicing donor site, which was one of the tag SNPs, is included in haplotypes #1, 2 and 3; the early polyadenylation allele of rs10954213 is in haplotypes #1, 4, 5 and 6; the insertion in exon 6 is in haplotypes #2, 3 and 6 (details in reference 1); and the 4x CGGGG allele in the IRF5 promoter is in haplotypes #4, 5 and 6 (current study).

In addition, we have correlated these six haplotypes with *IRF5* expression in four experiments with two collections of lymphoblastoid cell lines analyzed with microarrays[2]. This analysis showed some variability between experiments and probes but allowed to define a set of the most representative probes (from exons 2, 3, 5, 6, 7, 8, 9 and 3’UTR previous to rs10954213) and of multi-SNP models accounting for the changes in IRF5 expression. As part of this analysis, univariate linear regression coefficients for each of the six haplotypes defined above (coded as 0, 1 or 2 if absent, heterozygous or homozygous, respectively) and IRF5 expression were obtained. The mean of the coefficients corresponding to the four experiments were used in the current study to represent the level of expression corresponding to the haplotypes in table 3 of the main text. Expression was higher in the cells bearing the risk haplotype, #6. However, there was not clear distinction between neutral and protective haplotypes. In effect, the two haplotypes associated with the lowest expression were one of the protective haplotypes, #2, and a neutral haplotype, #3.

This distribution of haplotypes pertains to European subjects. Haplotype studies in patients with SLE of other ethnic groups have been less extensive but the available evidence indicates that there are important differences. In subjects from East Asia, there is convincing evidence of a protective haplotype that seems similar to the observed in Europeans[3,4]. However, no risk haplotype equivalent to #6 has been shown, and the most associated SNP in Japanese, rs41298401, is not polymorphic in Europeans (current study). In contrast, African American patients with SLE have a risk haplotype that is similar to #6 in Europeans but lack protective haplotypes and specifically rs729302 is not associated with SLE [5].

**References**

1. Ferreiro-Neira I, Calaza M, Alonso-Perez E, Marchini M, Scorza R, et al. (2007) Opposed independent effects and epistasis in the complex association of IRF5 to SLE. Genes Immun 8: 429-438.

2. Alonso-Perez E, Suarez-Gestal M, Calaza M, Kwan T, Majewski J, et al. (2011) Cis-regulation of IRF5 expression is unable to fully account for systemic lupus erythematosus association: analysis of multiple experiments with lymphoblastoid cell lines. Arthritis Res Ther 13: R80.

3. Kawasaki A, Kyogoku C, Ohashi J, Miyashita R, Hikami K, et al. (2008) Association of IRF5 polymorphisms with systemic lupus erythematosus in a Japanese population: support for a crucial role of intron 1 polymorphisms. Arthritis Rheum 58: 826-834.

4. Siu HO, Yang W, Lau CS, Chan TM, Wong RW, et al. (2008) Association of a haplotype of IRF5 gene with systemic lupus erythematosus in Chinese. J Rheumatol 35: 360-362.

5. Kelly JA, Kelley JM, Kaufman KM, Kilpatrick J, Bruner GR, et al. (2008) Interferon regulatory factor-5 is genetically associated with systemic lupus erythematosus in African Americans. Genes Immun 9: 187-194.

**Supplementary Note 2**

**RNA-seq study of the *IRF5* transcriptome and detection of potential *cis*-regulatory polymorphisms**

**Methods**

**RNA sequencing data:** We obtained the raw sequences for the transcriptome of 60 lymphoblastoid cell lines of the HapMap CEU collection from ArrayExpress under accession numbers E-MTAB-197 and E-MTAB-198 at <http://jungle.unige.ch/rnaseq_CEU60/>. These sequences correspond to a published RNA-seq study that used 37-bp paired end reads obtained in an Illumina Genome Analyzer II [1]. Data were available after QC filtering. The level of expression at the mRNA level was estimated as the count of reads covering a given sequence. To obtain this information, the sequencing reads were aligned to the human genome (Human March 2006 (NCBI36/hg18) Assembly) with the Burrows Wheeler Aligner (BWA) due to its proven ability to efficiently call indels[2]. We allowed five mismatches per read to include reads with different alleles at polymorphic sites. The reads not aligning to the reference genome could originate from exon-exon junctions. Therefore, they were aligned to a library of junctional sequences made with the 75 bases (or the length of the exon if < 75) in the 3’ end of each upstream exon, fused to the 75 bases (or the length of the exon) in the 5’ end of their downstream exon. The Integrated Genome Viewer (<http://broadinstitute.org/igv>) was used to visualize the data. Once aligned, reads mapping to each possible *IRF5* exon and its variants, as well as, their consecutive junctions were counted. This step was done with scripts developed in UNIX SHELL language delimiting the genomic region of interest.

**Genotypes from the 1000 Genomes data base:** We obtained the genotype data from the 1000 Genomes data base. We employed the pilot 1 genotype data of March 2010 release (<http://ftp.1000genomes.ebi.ac.uk/vol1/ftp/pilot_data/release/2010_3/pilot1/>) that was obtained with the same cell lines included in the RNA-seq study. Our analysis was restricted to the LD block that covers the entire *IRF5* and *TNPO3 loci* (chr7: 128350000-128495000; Human Mar. 2006 (NCBI36/hg18) Assembly). Only polymorphisms with a MAF > 5% were selected to a total of 234 SNPs and 29 indels.

**Statistical analysis:** Sequence read counts were transformed and normalized to the relative total number of reads in each cell line and to the exon length with the formula:

**
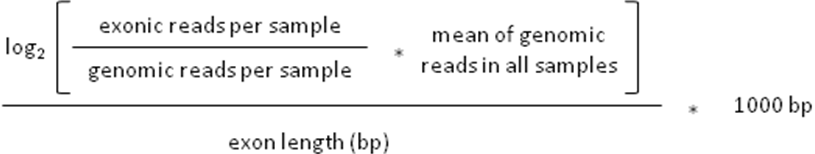
(1)**

This normalization was used to compare expression levels between exons and select those with correlated expression as representative of *IRF5* levels. The selected exons and the junctions between them were further analyzed by summarizing the counts of all of them, normalization to the relative sequencing depth for each cell line and transformation to log_2_ values, as detailed:


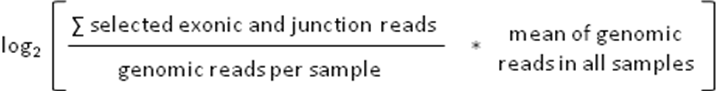
 (2)

Association of the *IRF5* expression phenotype defined in this way with the polymorphisms obtained from 1000 Genomes was determined with multiple linear regression. A genetic additive model was considered (with values 0, 1 and 2 for the AA, Aa and aa genotypes, respectively). The linear regression model was built following a forward entry process with the most associated polymorphism entering first and continuing until contribution to the model was not significant (*P* = 0.05).

**Results and discussion**

***Cis*-regulation of *IRF5* expression as evaluated with RNA-seq data:** We employed RNA-Seq data from HapMap CEU lymphoblastoid cell lines to determine *IRF5* expression levels. This methodology provides information that is complementary to the obtained with hybridization microarrays as the previously analyzed by us in relation with *IRF5*[3]. The advantages of RNA-seq include increased sensitivity for low copy transcripts, assessment of all splice-variants, discovery of new transcript variants and possibility of assessing allele-specific expression[4,5]. However, we did not attempted at this stage of analysis to extract all the potential information form the RNA-seq data. Our aim was to obtain with different technology information comparable to the provided by microarrays and consequently we restricted our analysis to the exon data showing the most uniform results for each cell line. A different technology is likely to produce results that are similar but not identical due to particular biases.

RNA-seq data for *IRF5* included sequence reads for its 9 exons and all the described exonic variants: four variants for exon 1, six variants for exon 6 and two variants for exon 9. They also included sequences spanning the boundaries of exons[4,5]. These sequences could originate from mature mRNA that has already undergone splicing. As our objective at this stage was to detect *cis*-regulatory polymorphisms affecting the most representative *IRF5* expression, we selected for further analysis the exon variants showing correlated expression. The expression counts used were log_2_ transformed and normalized in function of the relative depth of sequencing for the cell line and of the length of the specific exon variant (according to formula 1). The set of most correlated exons was made of exons 2 (128369361-128369566; coordinates from NCBI36/hg18), exon 3 (128373135-128373324), exon 6 variant corresponding to isoform V6[6] (128374611-128374825) and exon 7 (128375019-128375411). They showed a mean pairwise Pearson’s r = 0.94 among them. Once these 4 exons selected, the total amount of sequence reads included in them and their junctional reads (only exon 2-3 and exon 6-7 junctions were included) was normalized to the relative depth of sequencing by cell line and log_2_ transformed (according to formula 2). This quantity was used for the detection of *cis*-regulatory polymorphisms.

Multiple linear regression with a forward entry process was used to search the best model accounting for the representative expression data obtained above. Genotype information was obtained from the 1000 Genomes project that through resequencing has analyzed genetic variation in the same cell lines in which the RNA-seq data were obtained[7]. This source of genotype information is richer than the HapMap data specially for the detection of low frequency variants and of other polymorphisms than SNPs. In this way, it was possible to analyze 264 polymorphisms in the region of interest, whereas with similar criteria we have analyzed 109 in our previous study based in HapMap data. The best linear regression model obtained showed an adjusted r^2^ = 0.16 and a *P* value = 0.003. This model included two polymorphisms with significant contribution: rs11269962 (*P* value = 0.002) and rs11973360 (*P* value = 0.03). rs11269962 is located 2.2 Kb 5’ to *IRF5* (referenced to the start of exon 1 variant A) and it is an indel (14 bp) polymorphism that was not included in HapMap data. Its position and a good correlation with the SNP tagging *IRF5* protective haplotypes (r^2^ with rs729302 = 0.73) make of this SNP a possible *cis*-regulatory polymorphism of *IRF5* expression. On the contrary, the second polymorphism, rs11973360, is unlikely to act by itself regulating *IRF5* expression because it is located in a *TNPO3* intron. Therefore, it is probably associated with *IRF5* levels because of LD with other polymorphisms.

**References**

1. Montgomery SB, Sammeth M, Gutierrez-Arcelus M, Lach RP, Ingle C, et al. (2010) Transcriptome genetics using second generation sequencing in a Caucasian population. Nature 464: 773-777.

2. Li H, Durbin R (2010) Fast and accurate long-read alignment with Burrows-Wheeler transform. Bioinformatics 26: 589-595.

3. Alonso-Perez E, Suarez-Gestal M, Calaza M, Kwan T, Majewski J, et al. (2011) Cis-regulation of IRF5 expression is unable to fully account for systemic lupus erythematosus association: analysis of multiple experiments with lymphoblastoid cell lines. Arthritis Res Ther 13: R80.

4. Majewski J, Pastinen T (2011) The study of eQTL variations by RNA-seq: from SNPs to phenotypes. Trends Genet 27: 72-79.

5. Malone JH, Oliver B (2011) Microarrays, deep sequencing and the true measure of the transcriptome. BMC Biol 9: 34.

6. Mancl ME, Hu G, Sangster-Guity N, Olshalsky SL, Hoops K, et al. (2005) Two discrete promoters regulate the alternatively spliced human interferon regulatory factor-5 isoforms. Multiple isoforms with distinct cell type-specific expression, localization, regulation, and function. J Biol Chem 280: 21078-21090.

7. (2010) A map of human genome variation from population-scale sequencing. Nature 467: 1061-1073.
