# Supplementary material for: Identification of three new cis-regulatory IRF5 polymorphisms: in vitro studies
Source: Arthritis Res Ther. 2013 Aug 13;15(4):R82. doi: 10.1186/ar4262 (PMC3978921; doi:10.1186/ar4262)
Supplement: Additional file 3 — Figure S1 Western blot showing expression of IRF5 in WIL2 NS cells. Figure S2 Relationship of the r2 values between rs729302 and the other single-nucleotide polymorphisms included in our study obtained in our samples and in phase 1 of the 1000 Genomes projec. Figure S3 Functional analysis of the positive control: the CGGGG indel. Figure S4 Lack of difference between the two alleles of rs4728142 in electrophoretic mobility shift assay. [file ar4262-S3.DOC]

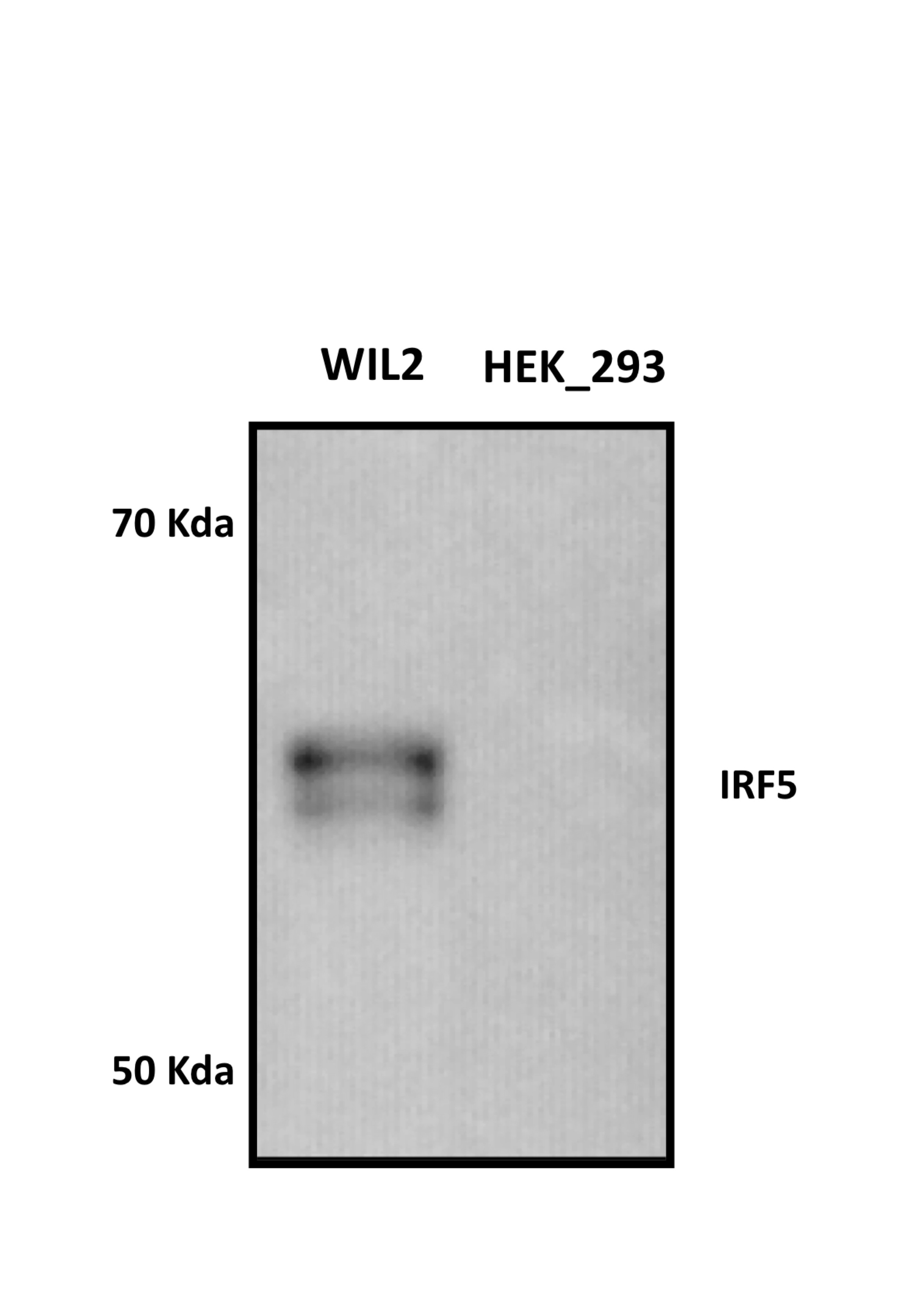


**Supplementary Figure 1: Western blot showing expression of IRF5 in WIL2 cells.** Total cell lystes were loaded in each lane (15 ug) WIL2 and HEK-293 cells (as negative controls). The IRF5 mouse monoclonal antibody 10T1 (Novus Biologicals) was used to reveal the protein bands. The two bands correspond to different IRF5 isoforms.

**Supplementary Figure 2: Relationship of the r^2^ between rs729302 and the other SNPs included in our estudy obtained in our samples and in the phase 1 of the 1000 Genomes project.** Forty four of the polymorphisms in our study were also present in the 379 European subject from the phase 1 of the 1000 Genomes project (october 31, 2012). The two sets of pairwise r^2^ values were highly correlated (Pearson r^2^ = 0.985).


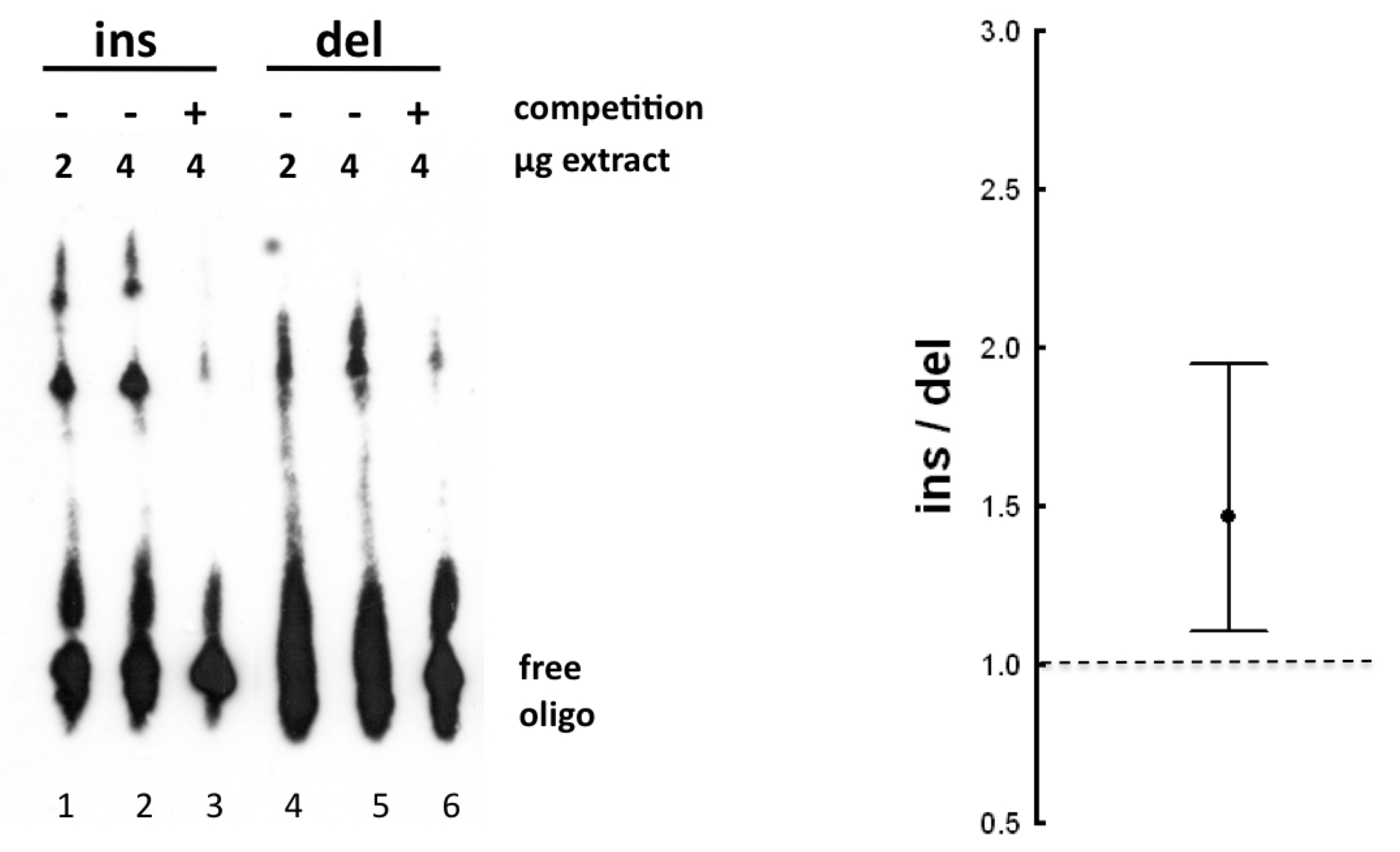


**Supplementary figure 3: Functional analysis of the positive control: the CGGGG indel.** A) The ins (4x) allele showed a slower band and a more intense middle band in the EMSA (lanes 1, 2) than the del (3x) allele (lanes 4,5). This difference was specific as shown by their absence after competition (lanes 3, 6). Experimental details are as in figure 3. B) The ins (4x) allele showed a higher expression of the *Firefly* luciferase reporter compared with the del (3x) allele. The geometric means and their 95 % confidence intervals of ratios between the luciferase signals of the WIL2 NS cells transfected with the two alleles are shown. * *P* < 0.05 by Wilcoxon matched-pairs test of five independent experiments.


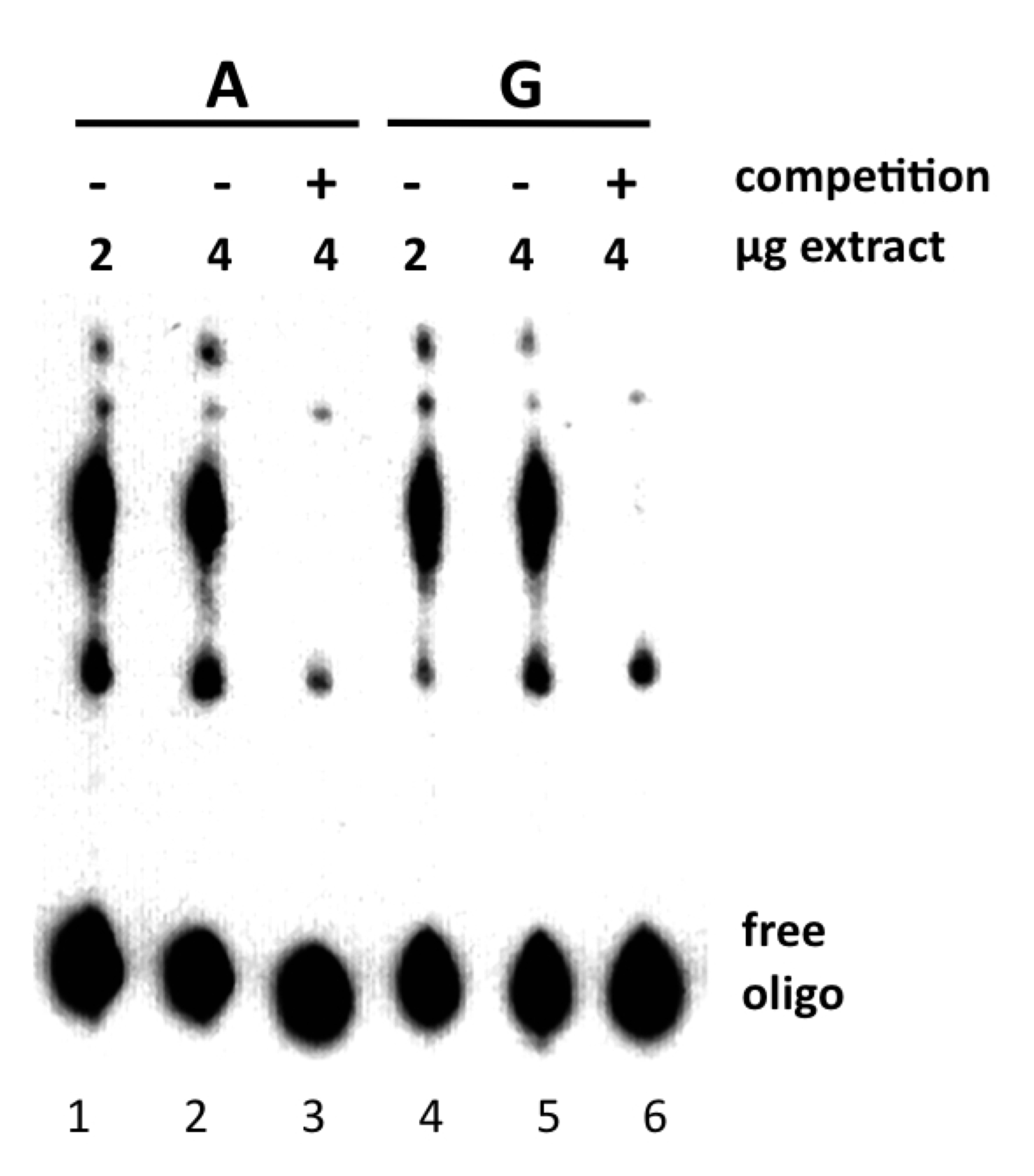


**Supplementary figure 4: Lack of difference between the two alleles of rs4728142 in EMSA.** The reported difference in intensity between the two alleles of this SNP [20] was not replicated in our analyses.
